# Investigation of Heavy Metal Analysis on Medicinal Plants Used for the Treatment of Skin Cancer by Traditional Practitioners in Pretoria

**DOI:** 10.1007/s12011-023-03701-4

**Published:** 2023-06-22

**Authors:** Oluwaseun Mary Oladeji, Boikanyo Genneyrolter Kopaopa, Liziwe Lizbeth Mugivhisa, Joshua Oluwole Olowoyo

**Affiliations:** 1 Department of Biology and Environmental Science, School of Science and Technology, Sefako Makgatho Health Sciences University, Ga-Rankuwa, South Africa; 2 Department of Health Science and The Water School, Florida Gulf Coast University, Fort Myers, USA

**Keywords:** Heavy metals, Medicinal plants, Skin cancer, ICP-MS, Health risk assessment

## Abstract

The use of medicinal plants for the treatment of diseases, including cancer, is acknowledged and accepted in many African nations. Heavy metal contamination of plant materials poses a potential health risk, particularly for populations that are already vulnerable. This study determines the levels of heavy metals in medicinal plant samples used for treatment of skin cancer and evaluate the health risk caused by heavy metals to the adult population in Pretoria, South Africa using inductively coupled plasma mass spectrometry (ICP-MS). The concentrations of metals were as follows; As (<0.2 – 1.04±0.026), Cd (0.02 ±0.00026 – 0.167±0.006), Pb (0.38 ±0.01 – 2.27±0.05), Cr (5.31±0.21– 26.9 ±3.96) mg/kg, and Hg which were lesser than 0.02 mg/kg. The mean concentrations of all analyzed heavy metals are above permissible limit except for Hg which are lower than the permissible limit. The Hazard Quotient (THQ) was less than 1 for all the heavy metals, suggesting that there are no obvious non-carcinogenic health risks associated with the consumption of these medicinal plants for now even though the prolonged use may result in health risks. The ingestion route was identified as the primary contributor to the overall risk by the health index (HI) values in the present study, which were more than 1, indicating that the combined effects of the heavy metal contaminants present in a particular herbal preparation pose health risk in the long term. Our findings support the need for close monitoring of potential heavy metal concentrations in medicinal plants given to patients from herbal shops.

## Introduction

Cancer is the second leading cause of death worldwide, after cardiovascular diseases, posing significant health risks [1]. The estimates of 14.1 million cases recorded in 2012 increased to 18.1 million new cases in 2018, and the death rate increased from 8.2 million to 9.6 million [2]. According to projections based on the Global Burden of Cancer study, the number of new cases of cancer will significantly increase to over 29 million by the year 2040 as a result of population growth and other social and economic factors [3]. Over 55% of all new cancer cases worldwide originated in developing nations [4]; of all the different types of cancer, a significant health issue on a global scale is skin disease [5, 6]. The skin is an outer organ that covers the body and performs a variety of vital tasks, such as fluid preservation, percutaneous absorption, controlling temperature, organ protection, maintaining body shape, and detoxifying the body by excreting toxins through sweat [7]. Skin cancer is one of the most common types of cancer in South Africa, making it a country with the second largest incidence of skin cancer in the world with approximately 20,000 cases and 700 deaths reported yearly [8, 9]. South Africans are extremely prone to skin cancer due to their year-round exposure to high ambient solar ultraviolet radiation (UV) and latitude (22–34°S) [10]. The frequent occurrence of skin issues makes them difficult to treat and has a significant impact on health. The causes of skin conditions show a strong link between a person’s health and sociocultural environment such as animal dander, certain foods, wool, or soaps, which provoke a reaction from the immune system, leading to symptoms like redness, pain, and itching [11]. Skin conditions can affect people of any age or gender. Due to poor healthcare management systems and a lack of resources to address the problems, this skin problem occurs [12].

In developing nations, herbal remedies are still frequently used as the first line of defense, and traditional medicine is frequently used to boost energy and strengthen the immune system, as well as to prevent and treat a variety of diseases [13]. Up to 80% of people in Africa’s rural areas rely on traditional medicine for their primary healthcare [14]. Like most African nations, South Africa has about 2600 plant species, with about 700 of them having known medical uses [15]. According to research, plants frequently contain other environmental toxins and microbes which produce biotoxins in addition to their harmful secondary metabolites, such as heavy metals, fungi, bacteria, pytotoxins insects, and predators [16]. In particular, heavy toxic metals are a concern because they are toxic to all living things and can be harmful when consumed. The herb’s root is a good absorber of heavy metals and metalloids [17], but, in spite of the presence of heavy metals in the growth substrate, they build up in the edible parts of the medicinal plant [18]. It has, however, been reported that medicinal products might be potentially life-threatening because they may possess some toxic heavy metals which are due to anthropogenic activities such as agricultural practices, mining, industrialization, natural activities such as rock weathering processes, cross-contamination during handling, and processing of medicinal plants after harvesting and during preparation [19]. Excess amounts of heavy metals have been reported to be extremely dangerous to human health, and research has shown that heavy metals rank high among main contaminants of leafy vegetables and medicinal plants [20]. As a result of the high prevalence of heavy metals in the environment, the residues of heavy metals may also reach and be assimilated into medicinal plants and medicinal herbs [21]. These heavy metals may have a negative impact on the ecology of the soil, the quality of agricultural products or production, the quality of ground water, and ultimately the health of living organisms through the oral intake or ingestion as is the case with medicinal plants.

Soil, climate, pollution, handling, atmosphere, and harvesting are just a few of the variables that may play a part in the poisoning of medicinal plants by trace metals [22]; heavy/toxic metals such as manganese (Mn), arsenic (As), cadmium (Cd), copper (Cu), lead (Pb), nickel (Ni), zinc (Zn), mercury (Hg), and chromium (Cr) if accumulate above the allowable level, cause harm to the blood’s composition, such as the liver, kidneys, lungs, and other vital organs, as well as impaired or diminished mental and central nervous function [23]. The World Health Organization recommends that some medicinal plants used as starting points for various medications be tested for pollutants like heavy/toxic metals, microbes, fungi, and pesticides [24]. In South Africa, the use of herbal mixtures is gaining popularity among various socioeconomic groups, particularly in rural communities where conventional or western medicine is not readily available, accessible, or affordable. Many herbal medicine users believe that herbal preparations or medicines are natural and have few side effects when compared to conventional drugs [25, 26]. Globally, numerous investigations have been carried out using ICP-MS to determine the metal content of medicinal plants [27–29]. Heavy metal contamination in South African medical plants was examined by [19] and [30]. The high concentrations of some toxic metals found in their findings raise serious questions about the quality of the herbal preparations, the source of the starting plant materials used in product manufacturing, and consumer safety. The South African Medicines Regulatory Authority (SAMRA) has made numerous efforts to legalize the practice of traditional South Africa medicine. However, issues such as medicinal product procurement, processing, and sale continue to be major worries. These procedure are essential for ensuring the safety and quality of herbal products [31]. Furthermore, WHO [32] states that trace element concentrations must be monitored in order to meet and improve quality assurance and safety. Pollution of the environment, including plants, continues to be a major problem due to the non-biodegradable nature of these pollutants, especially the toxic trace metals. The cultivation, harvesting, and processing of these medicinal plants may introduce pollutants at any stage, owing to the fact that Pretoria has been reported to have high concentrations of these toxic metals either in soil or water. The current study investigated the levels of heavy metals (As, Pb, Cd, Hg, Cu, Ni, Mn, Zn, and Cr) in five commonly medicinal plants intended for use and administered to cancer patients by traditional practitioners locally in Pretoria, South Africa. The purpose is to see if the levels of these toxic metals were within the acceptable limit and safe for human consumption, thereby predicting the overall effect on human health. Table 1 provides a general overview and economic significance of the medicinal plants prescribed by the traditional practitioners for treatment of skin cancer used in the current study.

**Table 1:** Medicinal plants prescribed by the traditional practitioners for the treatment of skin cancer

No	Local names	Scientific names	Uses	References
1	Mothokolo	*Carissa spinarum* L.	It used for treatment of skin diseases, cataracts, anemia, gastric ulcers, constipation, infertility, fever, asthma, myalgia, hypertension, asthma, and kidney complication	[33]
2	Vuma	*Myrothamus flabellifolia* Welw.	It used to moisturize skin, epilepsy, treat chest pains and mental disorders; it also has antiviral, antioxidant, antiarthritic, anticancer, antidiabetic, anti-inflammatory, antimicrobial, and antiulcer properties.	[34]
3	Phela	*Callilepis laureola* DC.	It is used to treat tapeworm, snakebite, infertility, as pregnancy tonics, kill maggots in cattle, and treat whooping cough.	[35]
4	Dabula valo	*Euclea crispa* Thunb.	It used to treat scabies, leprosy, diarrhea, gonorrhea, wound infections, and has antibacterial and antifungal activities.	[36]
5	Mohlakola	*Euclea divinorum* Hierm.	It used for the treatment of cancer, gastro-intestinal disturbances, cancer, toothache, leprosy, wound, miscarriage and jaundice arthritis, snakebites, headache, gonorrhea, and diarrhea	[37]

## Materials and Methods

### Study Area and Sampling

**Fig. 1:**
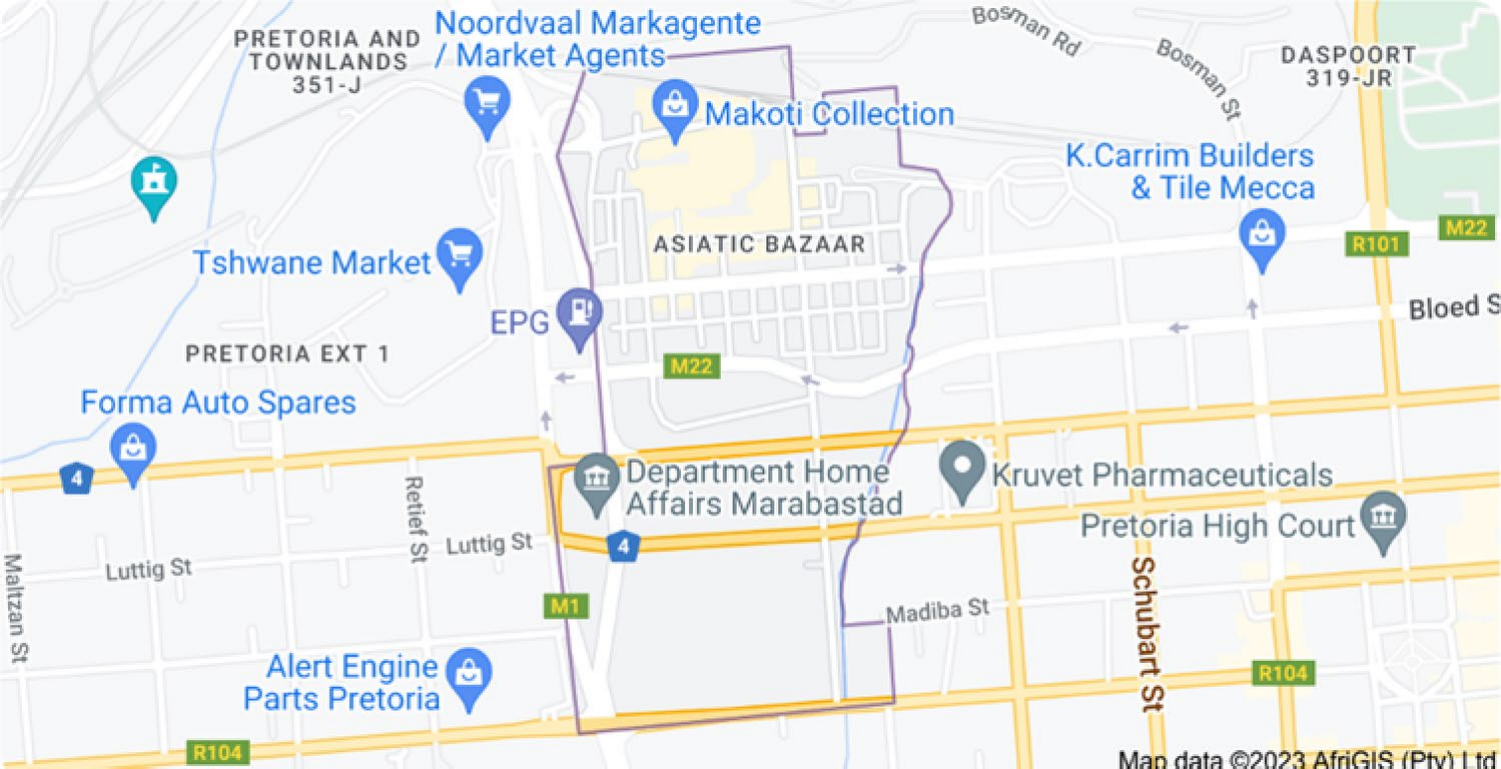
The geographical location of the sampling site where the medicinal plant samples were purchased. (https://www.google.com/search?q=marabastad+pretoria+map&source=lmns&bih=569&biw=1280&hl=en&sa=X&ved=2ahUKEwiLmZWyg8f9AhW9kScCHXtZBbEQ_AUoAHoECAEQAA)

The place where the medicinal plants were purchased is one of the business areas in Pretoria, South Africa and caters to people of different backgrounds visiting the city. It is a big commercial center in the city and generally a place where people go to buy different things with the impression that they are cheap when purchased from this place (Fig. 1). The high traffic of vehicles, both private and commercial, and different activities, including the burning of materials and tires in the open, characterize the area. In addition to this, there are some traditional healers in the area who practice their profession and are visited daily by people. The medicinal plants used for this study were purchased from the traditional healers in this area. Pretoria is located at a latitude of −25.731340 and a longitude of 28.218370, with the GPS coordinates of 25° 43′ 52.8240′ S and 28° 13′ 6.1320′ E. Medicinal plant species used, such as *Carissa spinarum* L., *Myrothamus flabellifolia* Welw., *Callilepis laureola* DC., *Euclea crispa* Thunb., and *Euclea divinorum* Hierm., were all purchased randomly depending on their availability from Muthi shops around the area in Pretoria, South Africa. The collected plants were botanically identified and authenticated by the African Centre for DNA Barcoding, in the Department of Botany and Plant Biotechnology, the University of Johannesburg.

### Sample Preparation

The medicinal plant samples used for this study were found dry and chopped into small pieces using a mortar and pestle in a laboratory, then transferred into a blender to crush them into fine powder. The ground samples were then packed in a Ziplock bag and keep in the refrigerator under −4 °C prior to the analysis.

#### Sample Acid Digestion

One gram of plant samples was weighed into a testing tube; 15 ml of nitric acid (HNO_3_) and 5ml of per-chloric acid (HClO_4_) were added to the sample. The mixtures were left overnight in a fume cupboard to dissolve into a homogenous mixture. The mixture was then placed on a stove at 90 °C, and the temperature was increased by 10 °C after every 2 min until it reached 170 °C. At 170 °C, drops of hydrogen peroxide (H_2_O_2_) were added into the mixture until the reaction was complete, which was indicated by the formation of white fume. The mixture was allowed to cool, and then deionized water was added to the volumetric flask until it reached 50ml. Subsequently, the solution was filtered through Whatman no. 42 filter paper. The resultant solutions were analyzed for heavy metals using inductively coupled plasma quadrupole mass spectrometry (ICP-MS).

#### Quality Control

A certified reference material of mixed polish herbs (INCT-MPH-2) from Poland was used to determine if the values obtained in the concentrations of the trace metals were accurate. The result for the CRM returned 90 – 95%. Spike and blank samples were also used to further determine the accuracy of the method used.

#### Statistical Analysis

The statistical analysis was carried out using Statistical Software Package for Social Sciences (SPSS) 28.0. Values obtained for the concentrations of trace metals in each of the medicinal plants were subjected to statistical analysis using the one-way analysis of variance (ANOVA) to determine if the differences in the concentrations were significant (*P*<0.05).

### Human Health Risk Assessment

On the basis of the estimated daily intake (EDI) of heavy metal, the hazard quotient (HQ), and the Hazard Index (HI), the risk of consuming heavy metal-contaminated herbal preparations to human health was assessed. To estimate the daily metals loading into the body system of a consumer with a given body weight, the daily intake dose (EDI) was calculated using [38, 39]. The following equation was used to calculate the value of EDI.

1 $$ED I=\frac{CS\ast IR\ast EF\ast ED}{BW\ast AT\ast 1000}$$

where EDI is the estimated daily intake of heavy metals ingested from a medicinal plant in mg/kg day, Cs is the concentration of heavy metal in medicinal plants measured in mg/kg, IR is the ingestion rate which is measured in mg/day, exposure frequency (EF) in days/year, ED is the exposure duration over years, BW is the body weight of the exposed individual in kg, AT is the time period over which the dose is averaged in days as seen in Table 2.

**Table 2:** Exposure parameters for the health risk assessment through various exposure pathways for plants

Parameter	Unit	Adult
Body weight	kg	70
Exposure frequency (EF)	days/year	350
Exposure duration (ED)	year	30
Ingestion rate (IR)	mg/day	100
Plant adherence factor (AF)	mg/cm^2^	0.07
Dermal absorption factor (ABS)	None	0.1
Dermal exposure ratio (FE)	None	0.61
Average time (AT): For carcinogens For Non-carcinogens	Days	365×70
	Days	365×ED

*US Environmental Protection Agency [40]

#### Hazard Quotient

In order to evaluate the non-carcinogenic risk that prolonged exposure to heavy metals from medicinal plants poses to people, the hazard quotient (HQ) method is used. If HQ is less than 1, there are no anticipated negative effects on health; if HQ is greater than 1, there may be adverse effects on health. According to the equation below, HQ is calculated as a percentage of the determined dose to the reference dose. The hazard quotient (HQ) is calculated as a ratio of average daily intake (EDI) to reference dose (*RfD*) [41, 42].

2 $$THQ=\frac{EDI}{RfD}$$

The following RFD were used: Cd (0.001), Cr (0.003), Mn (0.014), As (0.0003), Zn (0.3), Cu (0.04), Ni (0.02), Hg (0.0003), Pb (0.004) [43].

#### Hazard Index

The Hazard Index (HI) is a tool for assessing the total non-carcinogenic risk to human health posed by multiple heavy metals. Effects are additive when more than one pollutant is exposed. The following equation defines it as the total hazardous quotient (HQ) of all heavy metals. The HI was calculated as the sum of HQ (additional effects assumed):

3 $$HI=\sum HQ$$

Chronic risks are considered unlikely to occur if HI <1, whereas non-cancer risks are likely to happen if HI > 1.

## Result and Discussion

The profiles of medicinal plants used in the analysis were determined, and the results showed that the samples had variable compositions of each analyte metal with varying concentration ranges among various plant species. The mean concentration of heavy metals found in the five medicinal plants analyzed is presented in Table 3. The highest mean concentration for all the heavy metals was recorded for Mn with the concentration of 387.37±9.85 mg/kg in *Myrothamnus flabellifolius*, and the concentrations ranged from 16.73±0.65 mg/kg to 387.37±9.85 mg/kg. Mn is regarded as an important trace element that serves as an enzyme cofactor [44]. Although it is less toxic than any other metal, prolonged exposure to Mn dust and fumes can result in neurological disorders if its concentration exceeds 5 mg/m^3^ [45].

**Table 3:** Concentrations of heavy metals (mg/kg) in five medicinal plants used by traditional practitioners in Pretoria, South Africa

Plants names	As	Cd	Cr	Cu	Hg	Ni	Mn	Pb	Zn
*C. spinarum*	0.17±0.026	0.08 ± 0.01	18.07 ± 14.89	8.21 ± 1.31	< 0.2	7.55 ± 1.73	204.4 ± 30.20	1.63 ± 0.20	82.9 ± 9.50
*M. flabellifolius*	< 0.2	0.16 ± 0.00	9.93 ± 1.24	7.557 ± 0.21	< 0.2	18.97 ± 1.14	387.37 ± 9.85	2.27 ± 0.05	15.2 ± 1.04
*C. laureola*	< 0.2	0.02 ± 0.00	10.77 ± 0.51	7.20 ± 0.35	< 0.2	6.10 ± 1.03	48.87 ± 2.66	0.47 ± 0.02	24.37 ± 2.19
*E. crispa*	1.04±0.035	0.17 ± 0.001	26.9 ± 3.96	10.14 ± 0.63	< 0.2	18.8 ± 3.14	124.07 ± 5.49	2.25 ± 0.13	35.1 ± 3.32
*E. divinorum*	< 0.2	0.11 ± 0.001	5.31 ± 0.21	9.46 ± 0.93	< 0.2	11.50 ± 0.46	195.9 ± 0.98	0.49 ± 0.01	43.23 ± 1.95

The lowest mean concentration for all the heavy metals was recorded for Hg with the concentration <0.2mg/kg in all the medicinal plants. All of the medicinal plants analyzed for Hg were less than the detection limit of 0.2 mg/kg. In Canada, the limit of Hg in raw herbal material has been set at 0.2 mg/kg, while the limit in finished herbal products is 0.02 mg/day [46]. However, the WHO has suggested a 0.5 mg/kg limit for China and Singapore [46]. In the present study, the concentrations of Hg were within the permissible limit.

The concentration of Cu from all the selected medicinal plants ranged from 7.20 ±0.35 to 10.14±0.63 mg/kg. The highest mean concentration for Cu was recorded from *E*. *crispa*, while the lowest was recorded in *C*. *laureola*. The current finding showed that all traditional herbal products had Cu concentrations below the WHO-permitted limit (10 mg/kg) [47] except for *E. crispa*, which was above the acceptable limit of Cu in the plants. The metabolic process of humans depends on Cu. Copper controls a wide range of biological functions, including the production of energy, the formation of connective tissue, the synthesis of neurotransmitters, and the metabolism of iron. However, long-term exposure to a high copper concentration results in nasal mucosa irritation, vomiting, nausea, kidney, diarrhea, and liver damage [48, 49].

The concentrations of Ni ranged from 6.10±1.03 to 18.97±1.14mg/kg, and the highest concentration was recorded in *M*. *flabellifolius*, and the lowest concentration was recorded in *C*. *laureola*. The acceptable limit of Ni stipulated by the WHO for plants was 10 mg/kg, and according to the current study *M. flabellifolius*, *E. crispa*, and *E. divinorum* recorded high Ni concentrations that are above acceptable limit. Ni’s biological role in the human body is unclear. It is believed to be involved in the structure and function of proteins, but it is found in the highest concentrations in nucleic acids, particularly RNA [50]. The American Contact Dermatitis Society named nickel its 2008 allergen of the year, and its minimal risk level was set at 0.2 g/m^3^ for inhalation during 15–364 days. However, no limit has been established for food products [51, 52].

The mean concentration of Cr ranges from 5.31±0.21 to 18.07±14.89 mg/kg. The highest and the lowest concentrations were recorded in *C*. *spinarum* and *E*. *divinorum* plants respectively. Cr is toxic and fatal to humans. Cr is a non-essential element for plants, and it has no absorption pathway [53]. Plants cannot absorb this metal directly; instead, it accumulates via carrier ions such as sulfates or ferrous ions. Its toxicity interferes with germination, stunts growth by interfering with photosynthesis and other metabolic processes, and reduces total dry matter production. In contract to the WHO-recommended permissible limits for Cr (0.02 mg/kg), in the present study, the Cr concentrations exceed the maximum permissible limit in medicinal plants [54]. This may be the result of contamination during the harvesting and processing of medicinal plants. Owolabi et al. [55] and Adhikari et al. [56] reported high levels of chromium in medicinal plant from South African. Cr exposure can cause dermatitis, respiratory tract issues, lung cancer, and permanent nose damage [30].

Zn is an essential micronutrient in all biological systems [57]. The concentration levels of Zn ranged from 15.2±1.04 to 82.9±9.50mg/kg, with the highest and lowest levels occurring in *C*. *spinarum* and *C*. *laureola* plants. The mean concentrations of Zn in all the medicinal plants were above the acceptable limit of 0.60 mg/kg as recommended by WHO. In humans, high Zn can lead to metal poisoning and growth retardation, especially in young children [58]; in plants, it can stunt growth, damage leaves, and cause chlorosis, which lowers chlorophyll levels [59]. Cd levels ranged from 0.02±0.00 to 0.17±0.01 mg/kg. The highest and lowest concentrations were recorded from *E*. *crispa* and *C*. *laureola* plants respectively. The WHO maximum permissible limit of Cd in traditional herbal products is 0.02 mg/kg; Cd was detected in all the medicinal plant used in this study, but they were lower than the maximum permissible levels of elements in medicinal plant materials (Table 3) [60]. Similar findings have been reported in traditional medicine consumed in Thailand. High Cd intake is well known to be extremely toxic and carcinogenic. Lower doses can cause accumulation in the kidneys, which can damage the bones, the kidneys, and the lungs [30, 61].

One of the most toxic heavy metals is Pb, and prolonged exposure can result in impaired muscle coordination, digestive problems, kidney and brain damage, hearing and vision loss, and reproductive defects [62]. Pb bioaccumulates in biological tissues, putting patients at risk for chronic Pb toxicity if they use medicinal herbs containing even low concentrations of Pb for an extended period of time [63]. The concentration levels of Pb in the medicinal plants ranged from 0.47±0.02 to 2.27±0.05 mg/kg; the highest and lowest levels were recorded in *M*. *flabellifolius* and *C*. *laureola* plants respectively. Ibrahim et al. [64] reported various herbal medicinal plants sold in Saudi Arabia, with Pb levels below the WHO permissible limit (2 mg/kg) [26]; there results were in accordance with the present study. Previous studies in authors [30, 64, 65] reported Pb concentrations above the WHO maximum permissible limit in plants. The concentrations obtained for Pb in *M. flabellifolius* and *E. crispa* were above the acceptable limit in the present study. The As concentration in all the selected medicinal plants ranged from <0.2 to 1.04±0.03 mg/kg, and the highest As concentration was recorded in *E*. *crispa*. Although most herbs contain As, little is known about its biochemical function. One could argue that As is passively absorbed by plants through water flow. The established tolerance for As in plants is 0.50 mg/kg [66]; hence, *E. crispa* and *C. spinarum* are higher than the permissible limit. According to Martinez et al. [67], long-term exposure to As from water and food can cause cancer and skin lesions.

A number of variables, including soil pH, soil metal concentrations, plant species and variety, cation exchange capacity, organic matter content, and plant age, affect how much metal is taken up by plants [68]. The predominant factor, however, is the amount of the metal in the soil and, consequently, the current environmental circumstances [69]. As a result, medicinal plants for the preparation of herbal remedies should be harvested from unpolluted habitat.

### Health Risk Assessment of Heavy Metals Analyzed

One of the crucial tools for assessing health risk assessment is based on the estimated daily intake (EDI) of the heavy metal contaminant. It accounts for the exposure’s frequency, duration, and the body weight of the exposed people body weight. The estimated daily dietary intake (Table 4) generally determines the health risk posed by metal contamination. The mean EDI in the present study were as follows; Cu (1.8 × 10^−5^ mg/kg/day), Cr (2.4 × 10^−3^ mg/kg/day), Cu (1.42 × 10^−3^ mg/kg/day), Ni (2.08 × 10^−3^ mg/kg/day), Mn (3.2 × 10^−2^ mg/kg/day), Pb (2.37 × 10^−5^ mg/kg/day), and Zn (6.69 × 10^−3^ mg/kg/day). The results of this study revealed that the metals’ Estimated Daily Intake (EDI) in medicinal concoctions was lower than the oral reference dose (RfD). This suggests that consuming these medicinal concoctions poses no significant risk to public health.

**Table 4:** Estimated daily intake (mg/kg/day) according to the average concentration of each metal in each herbs item for adults

EDI (mg/kg/day)							
Plant names	Cd	Cr	Cu	Ni	Mn	Pb	Zn
*C. spinarum*	4.70 × 10^−5^	1.06 × 10^−2^	4.82 × 10^−3^	4.43 × 10^−3^	1.20 × 10^−1^	957 × 10^−4^	4.87 × 10^−2^
*M. flabellifolia*	9.39 × 10^−5^	5.83 × 10^−3^	4.44 × 10^−3^	1.11 × 10^−2^	2.27 × 10^−1^	1.33 × 10^−3^	8.92 × 10^−3^
*C. laureola*	1.17 × 10^−5^	6.32 × 10^−3^	4.23 × 10^−3^	3.58 × 10^−3^	2.87 × 10^−2^	2.76 × 10^−4^	1.43 × 10^−2^
*E. crispa*	9.98 × 10^−5^	1.58 × 10^−2^	5.95 × 10^−3^	1.10 × 10^−2^	7.28 × 10^−2^	1.32 × 10^−3^	2.06 × 10^−2^
*E. divinorum*	6.46 × 10^−5^	3.12 ×10^−3^	5.55 × 10^−3^	6.75 × 10^−3^	1.15 × 10^−1^	2.87 × 10^−4^	2.54 × 10^−2^

#### Non-carcinogenic Risk Assessment

Table 5 represents the non-carcinogenic hazard quotient (HQ) and non-carcinogenic Hazard Index (HI) calculated to be less than 1 for all the heavy metals found within the medicinal plants as shown in Table 5. The results of HQ indicated that for As, Cd, Pb, Cr, Ni, Hg, and Cu all were all less than 1, indicating that there is no health risk associated with consuming these herbal preparations because of these metals and the HI values for all the samples were more than 1, indicating that the combined effects of the heavy metal contaminants present in a particular herbal preparation pose health risk in the long term. Our study was similar to [70]; in their study, THQ value from individual herbs was less than 1, which was determined to be safe for human ingestion. [71–73] also stated in their study that their THQ was less than 1, stating that consumption of medicinal plants cannot pose any health risk to human health.

**Table 5:** HQ and HI (mg/kg/day) of each metal in each herb item for adults

THQ								
Plant names	Cd	Cr	Cu	Ni	Mn	Pb	Zn	HI
*C. spinarum*	4.69 × 10^−2^	3.53 × 10^−1^	1.20 × 10^−1^	2.21 × 10^−1^	8.57 × 10^−1^	2.39 × 10^−1^	1.62 × 10^−1^	2.00
*M. flabellifolia*	9.39 × 10^−2^	1.94 × 10^−1^	1.11 × 10^−1^	5.57 × 10^−1^	1.62	3.33 × 10^−1^	2.97 × 10^−2^	2.94
*C. laureola*	1.17 × 10^−2^	2.11 × 10^−1^	1.06 × 10^−1^	1.79 × 10^−1^	2.05 × 10^−1^	6.89 × 10^−2^	4.77 × 10^−2^	0.83
*E. crispa*	9.98 × 10^−2^	5.26 × 10^−1^	1.49 × 10^−1^	5.52 × 10^−1^	5.20 × 10^−1^	3.30 × 10^−1^	6.87 × 10^−2^	2.25
*E. divinorum*	6.46 × 10^−2^	1.04 × 10^−1^	1.39 × 10^−1^	3.37 × 10^−1^	8.21 × 10^−1^	7.19 × 10^−2^	8.46 × 10^−2^	1.62

## Conclusion

The findings indicated that concentrations of all the analyzed heavy metals in most of the medicinal plant samples used by traditional healers for the treatment of skin cancer were above the WHO permissible limit and may pose an additional health risk for consumers, especially those who reside in highly polluted environments. Nevertheless, an increase in environmental contamination and highly contaminated sites might be responsible for the accumulation of heavy metals in the plants, which can eventually end up in the human food chain. To ensure the safety of the consumers, it is essential to screen raw plant parts regularly to check the levels of pollutants and extracts before they are used. The current study’s report of trace metal concentrations in some samples emphasizes the need for more investigation into the security and assurance of the quality of South African herbal medicines, which are primarily based on the wild collection. The possible non-carcinogenic health risks associated with the consumption of the studied medicinal plants by adults was all less than 1, suggesting that there are no possible human health risks associated with consuming the studied medicinal plants at this stage. However, continuous consumption of these medicinal plants may result in bioaccumulation of these heavy metals in the body tissues of individuals which may lead to serious health problems. To avoid the buildup of pollutants and guarantee consistency in quality and efficacy, medicinal plants must be grown, harvested, and processed in a pollution-free environment.

## Data Availability

All provided in the manuscript.
